# Non-metastatic primary neuroendocrine neoplasms of the breast: a reference cancer center’s experience of a heterogenous entity

**DOI:** 10.3389/fendo.2024.1217495

**Published:** 2024-05-10

**Authors:** Mirosława Püsküllüoğlu, Aleksandra Grela-Wojewoda, Aleksandra Ambicka, Renata Pacholczak-Madej, Agnieszka Pietruszka, Anna Mucha-Małecka, Agnieszka Rudzińska, Marek Ziobro, Janusz Ryś, Jerzy W. Mituś

**Affiliations:** ^1^ Department of Clinical Oncology, Maria Sklodowska-Curie National Research Institute of Oncology, Kraków, Poland; ^2^ Department of Tumour Pathology, Maria Sklodowska-Curie National Research Institute of Oncology, Kraków, Poland; ^3^ Department of Anatomy, Jagiellonian University Medical College, Kraków, Poland; ^4^ Department of Radiotherapy, Maria Sklodowska-Curie National Research Institute of Oncology, Kraków Branch, Kraków, Poland; ^5^ Department of Surgical Oncology, Maria Sklodowska-Curie National Research Institute of Oncology, Kraków, Poland

**Keywords:** breast, breast cancer, neuroendocrine tumor, neuroendocrine carcinoma, breast neuroendocrine neoplasms, treatment

## Abstract

**Background:**

Primary neuroendocrine neoplasms of the breast (Br-NENs) are rare. The classification has been updated in recent years making interpretation of the data published challenging. It is unclear whether neuroendocrine differentiation is associated with poorer prognosis and what treatment approaches should be applied.

**Methods:**

The database for breast cancer patients treated between 2009 and 2022 at the Maria Sklodowska-Curie National Research Institute of Oncology Branch Krakow was explored to search for Br-NENs. Patients’ medical and pathological data were collected and analyzed.

**Results:**

We included 22 females with Br-NEN without metastases at the time of diagnosis. The median age was 64 years (range: 28-88), Of the cases, 18 were hormone receptor positive, all were HER-2 negative, the median Ki67 was 27% (10-100%). The median tumor size at the time of diagnosis was 29.5mm (7-75mm), 9 patients were N-positive. DCIS was present in 5 cases. Only one case was negative for chromogranin and synaptophysin staining, but data were missing for 4 cases. Nine patients received adjuvant chemotherapy, mainly based on anthracyclines and taxanes, while 16 received adjuvant hormonal therapy and 15 received postoperative radiotherapy. Radical surgery was performed in all patients, but two underwent suboptimal tumorectomy. One patient had local recurrence, three experienced metastatic disease, all involving the lungs, but these patients are still alive. The median follow-up was 96 months (8–153). Two patients died, with a follow up time of no recurrence >4 years. Our results were compared to twelve case series collecting clinical data on Br-NENs, with median patient number of 10.5 (range: 3-142).

**Conclusion:**

Br-NENs represent a heterogenous group of diseases, lacking data from prospective studies or clinical trials. There are no established treatment standards tailored for Br-NENs. Our patients’ cohort exhibited a favorable prognosis, potentially attributed to lower tumor stage and Ki67 index compared to other reported case series. We suggest that radical surgery and postoperative radiotherapy be administered akin to standard treatment for breast cancer of no special type. ESMO also advocates for this approach in systemic treatment, although we recommend considering platinum-based chemotherapy for patients with poorly differentiated Br-NENs exhibiting high Ki67.

## 1 Introduction

Breast cancer (BC) remains the leading cause of death for women worldwide (1). It is typically classified into several subtypes, including luminal A and B, Human epidermal growth factor receptor 2 (HER-2) positive and basal-like (with triple-negative being the most common among this subtype) based on the expression of estrogen receptor (ER), progesterone receptor (PR), HER-2 status, and Ki-67 status (2). Considering the considerable heterogeneity within BC, prognosis hinges on several key factors. These encompass the tumor’s subtype, disease stage determined by the tumor-node-metastasis (TNM) staging system or histological variants. The cornerstone of the treatment for non-metastatic BC is surgery supported by radiotherapy and various forms of systemic therapy (chemotherapy, hormonal therapy, immune checkpoint inhibitors and targeted treatment) (3–5).

The majority of breast malignancies arise from epithelial components and are classified as carcinomas (6). Ductal carcinoma (not-otherwise specified) is responsible for three-quarters of all cases, with lobular carcinoma accounting for 8 percent of cases. Other subtypes, such as mucinous, tubular, and medullary carcinomas, are less common, each comprising only around 1-2% of cases (6). Primary neuroendocrine breast neoplasms (Br-NEN) are extremely rare and heterogenous histotypes,accounting for less than 1% of all BCs. However, BCs with neuroendocrine differentiation seem to occur in higher number of BC cases (7, 8). Due to their diverse nature and rarity, prognostic factors as well as treatment guidelines remain controversial. Br-NENs are usually luminal (ER and/or PR) positive and HER-2 negative (7, 9). Data regarding prognosis are ambiguous, and it appears that few factors can influence it, including the level of expression of neuroendocrine markers, grade, stage, Ki-67 level, and presence of ER/PR (8).

There are several theories behind Br-NENs histogenesis, including cancer stem cells differentiating into both epithelial and neuroendocrine lineages, migration of cells from the neural crest to the mammary gland, or neoplastic transformation of primary breast neuroendocrine cells (7, 8, 10).

Neuroendocrine neoplasms (NENs) can arise in any part of the body since neuroendocrine cells can be found in almost all tissues and organs (8). The classification of neoplasms showing neuroendocrine (NE) differentiation has evolved over the years. Since the first introduction of NE carcinoma by the third edition of the World Health Organization (WHO) classification of breast tumors in 2003, the classification of NE cancers of the breast has undergone several revisions. In 2003, a category of NE tumors was defined as primary NE carcinomas exhibiting morphological features of NE tumors of gastrointestinal tract and lung with the expression of NE markers in >50% of cells (11). Four entities were distinguished in this group. It was also recognized that both breast carcinoma not otherwise specified and mucinous carcinoma can have NE differentiation. In the fourth edition of the WHO classification published in 2012, the threshold of >50% of cells immunoreactive to NE markers was removed from the definition of a category given the name of carcinomas with NE features (12). Notably, invasive breast carcinoma with NE differentiation appeared in this category with distinct ICD-O code (**Table 1**) and solid papillary carcinoma (formerly solid NE carcinoma) was defined, coded and included in a group of intraductal papillary lesions. The current fifth edition of the classification of breast tumors reflects the consensus of the International Agency for Research on Cancer (IARC) and the WHO to gather all tumors with predominant NE differentiation in one category of NENs – a category that applies to all anatomical locations. According to a uniform definition, NENs exhibit NE morphology characterized by various architectural patterns, including solid nests, trabeculae, cords, rarely ribbons, rosettes, papillae, insular patterns, and alveolar-like structures. These patterns are composed of cells that may be spindle-shaped, plasmacytoid, polygonal, and large. Additionally, NENs exhibit the presence of neurosecretory granules and display diffuse and uniform immunoreactivity for NE markers i.e., chromogranin proteins and/or synaptophysin. The category of NENs includes two subgroups: (1) neuroendocrine tumors (NETs) of low to intermediate grade and (2) neuroendocrine carcinomas (NECs) of high grade morphology. Breast NETs, unlike in other sites, are graded based on Nottingham grading system (NET G1 and NET G2, both defined as malignant), while NECs are further divided into small cell NEC (SCNEC) and large cell NEC (LCNEC), similarly to NECs of the lung (13–15). The histological types of breast tumors with NE differentiation distinguished in the last three editions of the WHO classification are presented in **Table 1** (15).

**Table 1:** The WHO classifications of breast tumors showing NE differentiation.

	Third edition, 2003		Fourth edition, 2012		Fifth edition, 2019	
	Entity	ICD-O code	Entity	ICD-O code	Entity	ICD-O code
**Neuroendocrine neoplasms**	Solid NE carcinoma	not provided	NA	NA	NA	NA
	NA	NA	NA	NA	NE tumor NOS	8240/3
	NA	NA	NA	NA	NE tumor, G1	8240/3
	Atypical carcinoid tumor	8249/3	NA	NA	NE tumor, G2	8249/3
	NA	NA	NE tumor, well-differentiated	8246/3	NE carcinoma NOS	8246/3
	Small cell/oat cell carcinoma	8041/3	NE carcinoma poorly, differentiated (small cell carcinoma)	8041/3	NE carcinoma, small cell	8041/3
	Large cell NE carcinoma	8013/3	NA	NA	NE carcinoma, large cell	8013/3
	NA	NA	Invasive breast carcinoma with NE differentiation	8574/3	NA	NA
**Invasive carcinoma of no special type**	NA	NA	NA	NA	Invasive ductal carcinoma with NE differentiation	8500/3
**Special subtypes of breast carcinoma**	Mucinous carcinoma (cellular/endocrine variant)	8480/3	Mucinous carcinoma (hypercellular/type B variant)	8480/3	Mucinous adenocarcinoma (hypercellular/type B variant)	8480/3
	NA	NA	Solid papillary carcinoma, invasive	8509/3	Solid papillary carcinoma with invasion	8509/3

G, grade; ICD-O, International Classification of Diseases for Oncology; NA, not applicable; NE, neuroendocrine; NOS, not otherwise specified; WHO, World Health Organization.

Apart from NENs, there are several histological types of breast carcinomas that may exhibit NE differentiation: (1) invasive carcinoma of no special type (NST)/invasive ductal carcinoma (10–30% of cases) as well as special types of breast carcinoma i.e. (2) hypercellular variant of mucinous adenocarcinoma (20% of cases) and (3) solid papillary carcinoma (over 70% of cases). The first type is diagnosed if NE morphology and NE markers expression are not uniform enough to meet the definition of NEN, whereas the other two – on the basis of their distinct morphologies as described in the WHO classification, regardless of the presence or absence of NE differentiation (13–15).

However, it has been postulated that the majority of breast cancers with NE differentiation are mixed neoplasms, consisting of a component of NEN and invasive carcinoma of conventional type. Therefore, the WHO recommends the following diagnoses: (1) mixed invasive carcinoma and NET/NEC if the NET/NEC component constitutes 10–90% of the tumor area, (2) NET/NEC if over 90% of the tumor presents with NET/NEC pattern and, finally, (3) invasive carcinoma if the NET/NEC element makes up less than 10% of the tumor (a comment on focal NE differentiation is optional in this case) (15).

The aim of the study was to evaluate clinical and pathological data of patients with primary breast neuroendocrine neoplasms treated at a reference Cancer Center in Krakow, Poland.

## 2 Materials and methods

### 2.1 Patients

Two independent researchers (JM and MP) identified patients with Br-NEN from the Maria Sklodowska-Curie National Research Institute of Oncology hospital registry system (2009-2022).

The inclusion criterion for the study was a diagnosis of Br-NEN based on a histopathology report. The diagnosis was based on the guidelines (WHO classification) applicable to the year of the patient’s diagnosis. Patients without an original pathological report were excluded from the study (information only mentioned in patient documentation) or doubts about the presence of a primary tumor in the breast (i.e., risk of metastasis to the breast from another location). Patients with coexisting active malignancies were also excluded. There were no restrictions on the age or sex of the patients.

Data regarding: age; clinical data (including: comorbidities, body mass index [BMI], menopausal status, family history, date of diagnosis; tumor location; clinical staging, Breast Cancer susceptibility gene [*BRCA*] mutation presence dates and types of treatment, recurrence dates and treatment; dissemination dates, location and treatment), histopathological data (including: histology, status of ER, PR, HER-2, Ki-67, chromogranin A and synaptophysin staining, presence of ductal carcinoma *in situ* [DCIS], tumor grade, pathological staging, version of WHO classification used), survival status, last visit date were gathered retrospectively.

The tumor was considered ER and PR positive if nuclear staining was observed in at least 1% of invasive tumor cells. HER-2 expression was evaluated in immunohistochemistry (ICH) with a score of 0-3, where 0 indicated no staining or weak-moderate incomplete staining in ≤10% of cells, 1 indicated weak and incomplete staining in >10% of cells, 2 indicated weak-moderate staining in >10% of cells or strong staining in <10% of cells, and 3 indicated strong complete membranous staining in 10% of cells. Cases with a HER-2 score of 2 underwent additional fluorescence *in situ* hybridization (FISH) analysis (16, 17).

### 2.2 Ethical considerations

The Maria Sklodowska-Curie National Research Institute of Oncology Branch Krakow Ethical Committee approval was obtained (decision no. 3/2023). Due to retrospective nature of the study written informed consents were not obtained from the patients as per Ethical Committee decision.

### 2.3 Statistical analysis

Statistical analyses were performed using Statistica v10.0. Elements of descriptive statistics were used, including proportions, means or medians (minimal, maximal), depending on the normality of the distribution. To check the normality of each continuous variable, both Shapiro-Wilk tests and histograms were used. Data were analyzed using either a chi-squared test or Fisher’s exact test, and statistical significance was defined as a p-value of less than 0.05.

## 3 Results

The study included 22 female patients with no male patient, and all of them had unilateral tumors. None of the patients was metastatic at the time of diagnosis. The median age at diagnosis was 64 years, spanning from 22 to 88 years, while the mean age was 61.6 years. Br-NENs accounted for less than 1% of the breast cancer patients in the Maria Sklodowska-Curie National Research Institute of Oncology Branch Krakow hospital registry system. Two additional patients were suspected of having metastatic Br-NEN at the time of diagnosis, but they were later diagnosed or were found to have a high probability of having a primary NE tumor in another location. These patients were not included into the study.

Considering changes in the WHO classification of breast tumors, the studied population was divided into the following histological subgroups: (1) carcinoids/well differentiated neuroendocrine tumors, (2) neuroendocrine carcinomas (small cell and not otherwise specified), (3) invasive ductal carcinomas/invasive carcinomas of no special type with neuroendocrine differentiation and (4) solid neuroendocrine carcinomas/solid papillary neuroendocrine carcinomas. (see **Table 2**).

**Table 2:** Primary neuroendocrine neoplasms of the breast characteristics.

Characteristics		N	N%
**Location - side**	Right breast	10	45.5
	Left breast	12	54.5
**Location - quadrant**	Upper outer	13	59.1
	Upper inner	3	13.6
	Lower outer	3	13.6
	Lower inner	1	4.5
	Central	3	13.6
**Br-NEN subtype**	Carcinoids/well differentiated neuroendocrine tumors	4	18.2
	Neuroendocrine carcinomas (small cell and not otherwise specified)	4	18.2
	Invasive ductal carcinomas/invasive carcinomas of no special type with neuroendocrine differentiation	10	45.5
	Solid neuroendocrine carcinomas/solid papillary carcinomas	4	18.2
**AJCC stage**	I	11	50.0
	II	7	31.8
	III	4	18.2
**pT**	1	5	22.7
	2	13	59.1
	3	2	9.1
	4	2	9.1
**Lymph node involvement**	Negative	13	59.1
	Positive	9	40.9
**Grade**	I	3	13.6
	II	8	36.4
	III	8	36.4
	No data	3	13.6
**DCIS presence**	Yes	5	22.7
	No*	17	77.3
**ER status**	Positive	18	81.8
	Negative	4	18.2
**PR status**	Positive	17	77.3
	Negative	5	22.7
**HER2**	Positive	0	0.0
	Negative**	22	100.0
**Synaptophysin**	Positive	17	77.3
	Negative	1	4.5
	No data	4	18.2
**Chromogranin A**	Positive	14	63.6
	Negative	4	18.2
	No data	4	18.2
WHO classification***	2003, third edition	8	36.4
	2012, fourth edition	9	40.9
	2019, fifth edition	5	22.7

* Or not mentioned the presence in hist-pat report.

** ICH: HER2-0 12; HER2-1; HER2 ICH 2 and FISH negative 7.

*** World Health Organization classification of breast tumors showing neuroendocrine differentiation applied

AJCC, American Joint Committee on Cancer, 8^th^ edition; Br-NEN, breast neuroendocrine neoplasms; ICH, immunohistochemistry; DCIS, ductal carcinoma in situ; ER, estrogen receptor; HER2, human epidermal growth factor receptor 2; N, regional lymph nodes; PR, progesterone receptor; pT, tumor.

The median Ki67 was 27% (range: 10-100%), with only three patients having a Ki67 of 50% or higher. Only one patient showed negative staining for both chromogranin A and synaptophysin, but her diagnosis was upheld after consultations.

Regarding the BC subtypes, 3 (13.6%) were luminal A, 12 (54.5%) were luminal B, 3 (13.6%) were luminal but could not be further classified, and 4 (18.2%) were triple-negative. The median tumor size at diagnosis was 29.5 mm (range: 7-75 mm), and further details can be found in **Table 2**, which includes histopathological information.

Only six patients underwent *BRCA* mutation testing, with one patient testing positive for BRCA1 mutation, but a family history of breast cancer was found in 6 (27.3%) cases.

Almost all regimens were based on anthracycline and subsequent taxanes. None of the four patients who received chemotherapy based on anthracyclines or taxanes in the neoadjuvant setting experienced tumor response. In two cases, planned chemotherapy was terminated due to suspicion of clinical progression, and the patients underwent earlier surgery. Two patients with Ki67 levels above 80% received platinum-based regimens as part of their systemic treatment, including one patient treated in the neoadjuvant setting who exhibited a particularly good response and was also administered adjuvant capecitabine. Sixteen patients had adjuvant hormonal therapy, and 15 had postoperative radiotherapy, following the prevailing treatment guidelines at the time. All patients underwent radical surgery, but two had suboptimal tumor removal. One of these patients had local recurrence 26 months after tumorectomy. Initially, the patient chose to avoid radiotherapy and systemic therapy but later underwent mastectomy with 5 years of adjuvant hormonal therapy and is now under follow-up for 7 years.

Three patients experienced metastatic lung disease (one with coexisting liver metastases, one with concurrent bone metastases and a suspicion of liver metastases) 2, 4, and 7 years after the initial diagnosis. At the time of closing the database (February 2023), they were still alive with a follow-up of 3, 7, and 10 years, respectively, and were receiving multiple lines of treatment including hormonal therapy +/- cyclin-dependent kinases (CDK) 4/6 inhibitors, and chemotherapy in the palliative setting. The median follow-up for the entire population was 96 months (range: 8-153 months). Two patients died without recurrence after more than 4 years of follow-up; they were both aged over 80 years and had significant comorbidities

Our study did not find any significant correlations between age, menopausal status, BMI, tumor size, grade, lymph node status, and the presence of distant metastasis or recurrence. Similarly, no correlations were observed between BMI, family history, menopausal status, and the tumor stage or lymph node involvement.

**Figure 1:**
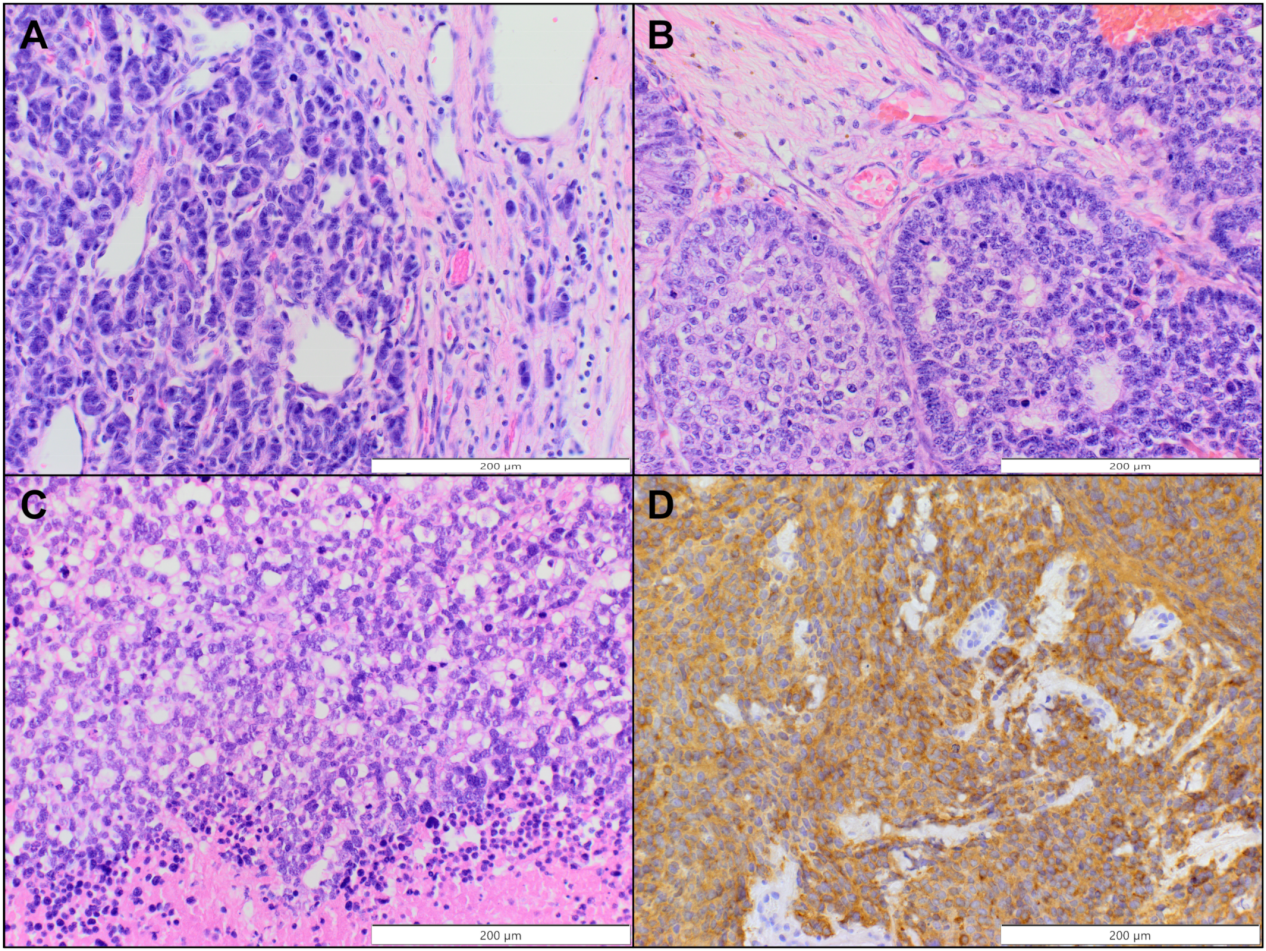
Selected primary neuroendocrine neoplasms of the breast. **(A, B)** Invasive carcinoma of no special type with neuroendocrine differentiation (H&E, original magnification 200x). **(C)** Small cell carcinoma (H&E, original magnification 200x). **(D)** Positive immunohistochemical staining for synaptophysin (case presented in C, original magnification 200x).

Pathological and clinical data regarding patients are presented in **Table 2** and **Table 3**. **Figure 1** presents stainings for selected primary neuroendocrine neoplasms of the breast.

**Table 3:** Patients’ clinical characteristics.

Characteristics		N	N %
**Important comorbidities**	Yes	15	68.2
	No	7	31.8
**Family history of breast cancer**	Present	6	27.3
	Absent		0.0
**Menopausal status**	Postmenopausal	16	72.7
	Premenopausal	6	27.3
**Way of tumor detection**	Noted by the patient	15	68.2
	Screening program/accidental finding on imaging tests	7	31.8
**Surgery - tumor**	Breast conserving surgery*	16	72.7
	Mastectomy	4	18.2
	Skin-sparing mastectomy	1	4.5
	No data	1	4.5
**Surgery - lymph nodes**	Sentinel lymph node procedure	13	59.1
	Axillary lymph node dissection	5	22.7
	No data about type of procedure	2	9.1
	Not performed	2	9.1
**Radiation therapy**	Yes	15	68.2
	No	6	27.3
	No data	1	4.5
**Chemotherapy**	Yes	8	36.4
	No	14	63.6
**Hormonal therapy**	Yes**	16	72.7
	No	4	18.2

*Including 2 cases of suboptimal tumorectomy (1 due to comorbidities, one due to patient’s decision).

**All luminal-type patients.

## 4 Discussion

(11, 12)As our patients were diagnosed between 2009 and 2022 the classification varied depending on the year of diagnosis making it extremely challenging to classify the presented cohort when assessed retrospectively (**Table 2**). Therefore, after careful discussion between pathologists and clinicians, we decided to keep patients in our database who were initially diagnosed with Br-NENs but may not fit the Br-NENs category in the later classification.

Other authors have also encountered difficulties in defining the types of NE malignancies included in their case series and in determining the nomenclature. Clinical data on Br-NENs were collected in only twelve case series, with a median of 10.5 patients (range: 3-142) per series, which are summarized in **Table 4**. The published case reports are presented in the **Supplementary Materials** (**Table S1**).

**Table 4:** Summary of presented in the literature case series and retrospective cohort studies of neuroendocrine carcinomas of the breast with selected immunohistochemical features, applied treatment modalities, and outcome.

Age*/Sex	Histopathologic features	IHC	N+	M+	Surgical Treatment	Systemic treatment	Outcome	Ref
142 cases: 64 (IQR 26-99)/ 139W, 3M	Neuroendocrine differentiation >50% of the tumor cells express neuroendocrine markers	ER(+), n=77,54%; PR(+), n=54,37%; HER2 no data	n=40, 28.2%	n=39, 24%	No surgery n=33,23.2%; Surgery n=109, 76.8%	No data	mOS 26 mo (IQR 12-48)	(18)
74 cases: 61 (IQR 29-81)/72W, 2M	Primary NEC of the breast	ER(+) n=70, 92%; PR(+) n=59, 69%; HER2 (-) n=67, 90%;	n=31, 42%	n=6, 8%	Mastectomy n=20, 27%; Partial mastectomy n=13, 18%	NCT n=17, 23%; Adjuvant chemotherapy n=24, 31%; Adjuvant endocrine therapy n=44, 59%	Local mRFS 177 mo; Distant mRFS 73 mo	(19)
7 cases: 48 (IQR 36-63)/W	Invasive breast cancer NST with neuroendocrine features n=2, 28.6%; Neuroendocrine neoplasms n=5, 71.4%	ER(+) n=5, 71%; PR(+) n=5, 71%; HER2(-) n=7, 100%; Syn(+) n=6, 86%; Chr(+) n=6, 86%;	n=4, 57%	At diagnosis n=4, 57%, overall n=7, 100%	Wide local excision or mastectomy n=3, 43%	Palliative chemotherapy in n=7,100%: 1^st^ line CBDDP+V16 n=4, 57% Adjuvant endocrine therapy n=4, 57%	mOS 31.8 mo (IQR 3.7–108.6)	(20)
12 cases: 66.5 (IQR 43-79)/W	Primary NEC of the breast	ER(+) n=11, 91%; PR(+) n=11, 91%; Syn (+) n=11, 91%; Chr (+) n=5, 41%; NSE(+) n=12,100%	n=1, 8%	No	Radical mastectomy with axillary lymph node dissection n=6, 50%; Lumpectomy n=6, 50%	No data	mRFS 24 mo	(21)
9 cases: 51 (IQR:46-62)/W	Small cell carcinoma of the breast	ER(+) n=5, 56%; PR(+) n=5, 56%; HER2(-) n=9, 100%; Syn(+) n=6, 66%; Chr(+) n=6, 66%;	n=4, 44%	In 2 cases (22%) after 11 and 32 mo	Mastectomy n=3, 33%; Lumpectomy n=6, 66%; Axillary dissection n=9, 89%	Adjuvant chemotherapy n=7, 78%; Adjuvant endocrine therapy TAM n=2, 22%	RFS 3-35 mo	(22)
3 cases: 60 (IQR:46-61)/W	Small cell NEC	ER(+) n=0; PR(+) n=0; HER2(-) n=3, 100%; Syn(+) n=2, 66%; Chr(+) n=3,100%; NSE(+) n=3, 100%; CK7(+) n=3, 100%;	No	No	Lumpectomy n=3, 100%	Adjuvant chemotherapy DDP + VP16 n=3, 100%	2 cases RFS 48 and 6 mo; 1 case OS 20 mo	(23)
24 cases: 47.8 (IQR 28-74)/W	Neuroendocrine DCIS n=9, 37.5%; Microinvasive NEC n=7, 29.2%; Invasive NEC n=8, 33.3%;	ER(+) n=0; PR(+) n=0; HER2(-) n=24,100%; Syn(+) n=24,100%; Chr(+) n=24,100%;	No	No	Lumpectomy n=15, 62%; Radical mastectomy n=9, 38%	No data	mRFS 83.7 mo	(24)
4 cases: 52.5 (IQR:41-66.5)/W	Oat cell NEC of the breast	ER(+) n=1,25%; PR(+) n=1, 25%; Syn(+) n=2, 50%; Chr(+) n=1, 25%; NSE(+), n=3, 75%	n=3, 75%	No	Radical mastectomy n=4, 100%	Adjuvant chemotherapy streptozotocin followed by palliative chemotherapy CMF n=1, 25%; Adjuvant endocrine therapy TAM n=1, 25%	1 case RFS 44 mo; 2 cases OS 14 and 15 mo; 1 case died of other cause after 9 mo	(25)
5 cases: 67 (IQR:53-73)/W	Well/moderately differentiated NEC of the breast n=3, 60%; Small cell NEC of the breast n=2, 40%	ER(+) n=3, 60%; PR(+) n=4, 80%; HER2(-) n=5, 100%; Syn (+) n=5, 100%; Chr(+) n=4 80%;	n=4, 80%	n=2, 40%	Partial mastectomy with axillary lymph node dissection n=3, 60%	Palliative chemotherapy 1^st^ line CBDDP/DDP+V16 n=2, 40%; Adjuvant endocrine therapy TAM n=1, 20%	3 cases RFS:12,25 and 129 mo; 2 cases OS 8 mo and 10 mo	(26)
56 cases: 60 (IQR:34-81)/W	Various entities	ER(+) n=56,100%; PR(+) n=48, 86%; HER2(-) n=48, 86%; Syn(+) n=50, 89%; Chr(+) n=41, 73%;	n=20, 36%	n=3, 5%	Mastectomy and SLND n=21, 38%; Partial mastectomy and SLND n=31, 55%	NCT n=4, 7.1%; Adjuvant endocrine therapy TAM n=8, 14%	Alive n=54, 96%; Dead n=2, 4%	(27)
4 cases: 58 (IQR:50-65)	Primary NEC of the breast	ER(+) n=4, 100%; PR(+) n=3, 75%; HER2(-) n=4, 100%; Syn(+) n=2, 50%; Chr(+) n=3, 75%;	n=3, 75%	n=0	Radical mastectomy n=4, 100%	Adjuvant chemotherapy CAF n=4, 100%; Adjuvant endocrine therapy n=4, 100%;	mRFS 27 mo (IQR: 7-48)	(28)
5 cases: 55 (IQR:52-65)/W	Primary NEC of the breast	ER(+) n=5, 100%; PR(+) n=4, 80%; Syn(+) n=4, 80%; Chr(+) n=3, 60%; NSE(+) n=4, 80%	n=4, 80%	n=0	Quadrantectomy n=4, 80% with SLND in 2 cases and axillary lymph node dissection in 2 cases; Mastectomy with axillary lymph node dissection in 1 case (20%)	Adjuvant chemotherapy DDP + VP16 n=3, 60%; Adjuvant endocrine therapy TAM n=5, 100%	Alive n=4, 80%; Dead n=1, 20%	(29)

*The age is presented as medians and IQR.

CAF- cyclophosphamide + adriamycin + 5-Flurouracil; CBDDP- carboplatin, CEF- cyclophosphamide + epirubicin + fluorouracil, Chr- chromogranin, CK- cytokeratin, CMF-cyclophosphamide+ methotrexate + 5-Fluorouracyl, DCIS- ductal carcinoma in situ, DDP-cisplatin, EC- epirubicin + cyclophosphamide, ER- estrogen receptor, HER2- human epidermal growth factor receptor 2, IHC- immunohistochemistry, IQR -interquartile range, M+ presence of distance metastases, M-men, mOS/OS- median/overall survival, mo-months, mRFS- median/relapse-free survival, n- number of cases, N+ presence of metastases in regional lymph nodes, NCT- neoadjuvant chemotherapy, NEC- neuroendocrine carcinoma, NSE- neuron specific enolase, Syn- synaptophysin, PR- progesterone receptor, PXL- paclitaxel, Ref- reference, SLNB- sentinel lymph node biopsy, TAM- tamoxifen, TC- docetaxel + cyclophosphamide, V16- etoposide, W-women, y-years.

The primary concern when making a differential diagnosis is the possibility of a metastatic neuroendocrine tumor originating from an extramammary location (30). Although metastases from NE tumors to the breast are rare, we have identified two cases with such suspicion in our registry. Other authors have also reported similar situations when collecting Br-NENs patients’ cohorts (27).

In a case series by Singh et al. (not included in **Table 4**, due to lack of clinical data gathered in that paper) half of the patients had positive HER-2 status, while in our study and other case series, all cases were negative (22, 24). However, our data confirm findings from other cohorts in terms of majority of tumors being ER or PR positive (29, 31). Published data also included cases of Br-NENs negative for synaptophysin or/and chromogranin A staining, similar to one of our patients (21, 22, 24, 31). Interestingly, when compared with other authors (e.g (27).) the percentage of luminal A subtypes is much lower in our cohort, although the prognosis in our patients was favorable. The median Ki67 index was 27%,with only three patients having a Ki67 of 50% or higher. In a cohort of metastatic Br-NENs median Ki67 was 50% (20).

As indicated by other researchers, the majority of patients diagnosed with Br-NEN are postmenopausal women in their fifth to seventh decade of life, with most cases occurring in those aged over 60 (8, 21). Our group seem to confirm that characteristic. Br-NEN rarely affects males, in our cohort we had no male patient. Age and family history are currently considered the primary risk factors for Br-NEN, similar to non-neuroendocrine BC (8, 9, 18, 19). In the largest study with over 140 Chinese and American patients with neuroendocrine breast carcinomas the tumor grade and Ki-67 levels played a crucial role as prognostic factors for disease-free survival (DFS), while the age and ER status were significant prognostic factors for overall survival (OS) (9).

Almost one quarter of our patients had a family history of BC. However, due to the small number of tested patients, we cannot provide the percentage of those with a *BRCA* mutation. In other studies, the percentage of patients with a family history of BC (if assessed) was also high, around 20% (27). The majority of our patients detected the tumor themselves (see **Table 3**). Interestingly, Kawasaki et al. suggest that neuroendocrine BC are more prone to cause nipple discharge than other breast malignancies (24).

There are currently no clinical trials or prospective studies available regarding the treatment of Br-NENs. However, data from a few case series published thus far are presented in **Table 4**, and a compilation of all published case series up to January 2023 can be found in the **Supplementary Materials** (**Table S1**).

The comprehensive management of BC is determined by cancer stage, biological markers, tumor histology and factors related to patients: performance status, coexistence of comorbidities or menopausal status. Treatment guidelines for non-metastatic BC consider staging and BC subtypes, but histological variants are neglected (3). While rare pure and mixed histological subtypes other than ductal and lobular carcinomas are not individually predictive, they may have prognostic value when combined with data on staging, grading, and biomarker status (32). Surgical treatment, radiotherapy, and systemic therapy based on classical prognostic and predictive factors are the current standard of care (13, 33–36).

Therapeutic decisions regarding adjuvant systemic treatment take into account tumor size, lymph node involvement, Ki-67, and biological features (ER/PR and HER-2 status). Endocrine therapy is indicated in all ER/PR positive patients. (Neo)adjuvant chemotherapy in Br-NENs is used according to standard guidelines and is typical and based on anthracycline/taxanes regimens (13, 36, 37). Poorly differentiated small or large cells cancers can be treated with platinum/etoposide regimens (33, 36, 38). In the study group, 2 patients received perioperative chemotherapy with cisplatin/etoposide (one in neoadjuvant setting). In both cases, these were patients with triple-negative breast cancer, cT3N2, with grade 3, and Ki67≥ 80%. Both patients remain in a follow-up without recurrence. Chemotherapy with platinum and etoposide was also applied to a group of three patients described by Adegbola et al. (23). In our case series, other patients received anthracyclines/taxanes (if required and if allowed by comorbidities). However, poor responses were observed for all 4 patients, when administered in the neoadjuvant setting. Only 8 published retrospective patient case series provide data on systemic treatment (**Table 4**), including one involving patients treated more than 30 years ago. Wei et al. demonstrated statistically non-significant shorter survival in patients with breast NE carcinoma who received chemotherapy as a part of their treatment, however they do not specify the regiments (19). In contrary, Shin et al., in their small case series, reported good outcomes after applying chemotherapy to seven out of nine patients treated in their cancer center (also without specifying the chemotherapy regimens) (22).

In our study, 18 cases presented with positive ER/PR status. Sixteen received hormone therapy (tamoxifen, aromatase inhibitor, gonadotropin-releasing hormone analogue) in the adjuvant setting for at least 5 years, with some cases extending therapy beyond this duration. One patient lacked this data in the documentation, and another did not consent hormonal agents treatment. Other studies also show improved survival trends in patients receiving hormonal agents (19). There were no patients with HER-2 overexpression in our study group (13). In HER-2-positive cases, chemotherapy and anti-HER-2 therapy are employed based on cancer stage and risk factors (36, 39, 40).

In patients with positive somatostatin receptors (SSR) the treatment with somatostatin analogues (SSAs) or receptor radionuclide therapy (PRRT) can be considered, although the data are scarce (8, 13). Study by Vranic et al. identified potential targets for novel therapies on a sample of 20 neuroendocrine cancers (41). Authors detected expression of Trophoblast cell surface antigen 2 (TROP-2), Folate receptor 1 (FOLR1) and Trimethylated Lys-36 of histone 3 (H3K36Me3) – targets for sacituzumab govitecan, farletuzumab soravtansine and histone deacetylase inhibitors respectively (41). Trevisi et al. suggested Phosphatidylinositol-4,5-Bisphosphate 3-Kinase Catalytic Subunit Alpha (PIK3CA) as a potential target for alpelisib in Br-NENs (42). Another promising strategy is the use of immune checkpoint inhibitors. However, prospective studies are still needed to fully understand their effectiveness in treating Br-NENs and other BC malignancies (5, 13, 43).

Most of our patients received postoperative radiotherapy. There are no specific guidelines for the use of radiotherapy in patients with Br-NENs. Literature data suggest that postoperative radiotherapy should be performed similarly to that of invasive BC-NST and such radiotherapy was utilized in our patients (7, 13, 44–46). The decision on radiotherapy depends on thetumor size, clinical stage and type of surgery performed. Wei et al. presented the results of treatment for 74 patients with NEC BC who were managed as BC-NST (19). In the analyzed group, patients who received radiotherapy had longer OS (median 156 vs. 88 months) and distant recurrence-free survival (DRFS) (median 138 vs. 80 months) than those who did not receive it. However, the differences were not statistically significant (19). This may be attributed to the small size of the analyzed group, resulting from the limited number of patients with this diagnosis. Hare et al., in a cohort of 199 patients with primary small cell carcinomas of the breast (SCCB), did not observe differences in median OS in patients treated with and without radiotherapy, both in the groups of locally and locoregionally advanced cancers (47) (this study was not included in **Table 4** as it lacked numerous other patients’ data such as status of the receptors, type of chemotherapy or surgery applied). The role of radiotherapy remains a subject of further research, and a better understanding of the biology of these rare cancers may contribute to the development of optimal therapeutic strategies.

Surgery is the recommended treatment for patients with resectable Br-NEN. It is important to distinguish between primary and metastatic lesion in the breast (13, 48). In our presented group, 16 (73%) of the patients underwent breast-conserving surgery (BCS), and 13 (60%) of them had a sentinel lymph node biopsy (with only one requiring subsequent ALND). One patient, 26 months after primary BCS, underwent surgery due to a local recurrence. A second breast conserving treatment was performed, with a sentinel lymph node biopsy, followed by radiation therapy, resulting in a successful outcome. There is evidence to suggest that the lack of surgical treatment leads to a worse outcome (shorter OS) in Br-NENs (18, 35, 49). However, due to the rarity of Br-NEN, there is limited evidence on the optimal extent of resection for primary early-stage cases (50). Current guidelines for Br-NEN treatment follow those for non-neuroendocrine carcinomas of the breast, which include a variety of surgical treatment options specified by NCCN guidelines for different types of breast cancer (35, 37). Case reports and case series have demonstrated that standard surgical strategies used for typical types of breast cancer can be successful for Br-NEN. BCS is an established treatment for typical types of BC, but there is limited or insufficient evidence to support its use for Br-NEN. Mastectomy, presented by some authors as preferred surgical treatment for early-stage Br-NEN due to its aggressive nature (51), was only applied in 4 cases in our study (5 when including sub-cutaneous mastectomy), with the majority of patients receiving BCS. Axillary dissection is recommended for cases with confirmed lymph node metastasis or positive sentinel lymph nodes, following standard guidelines (37). In specific cases, the surgical removal of potentially radically resectable liver metastases can lead to extended survival (52). However, liver surgery should only be pursued if R0 resection is achievable and there is no evidence of extrahepatic disease (52).

Some researchers claim that Br-NENs, particularly small-cell carcinoma, are associated with poor prognosis among rare histological subtypes of breast cancer (19, 31). NECs are typically diagnosed at a more advanced stage with larger tumor size and higher frequency of metastasis to regional lymph nodes than non-neuroendocrine breast carcinomas (53). In our case series, except for two cases (death from other causes in patients without recurrence/dissemination and older than 80 years), all patients are alive in the last follow-up (median follow-up 96 months; 8-153 months). Our group’s prognosis for patients with Br-NENs is not as poor as suggested. Similar results were reported for a series of seven patients by Shin et al. (22). It is recommended to conduct long-term monitoring as Br-NEN has the ability to spread to various locations, even several years after the primary tumor treatment. Possible metastatic sites may include the lungs, liver, bones, pancreas, soft tissues, and brain. In our cohort, metastases were found in the lungs, liver, and bones. Other authors have also not confirmed significant correlations between factors such as tumor size, lymph node involvement, grading, ER/PR, or Ki67 status, and the presence of metastases (35, 52).

It is important to note that patients requiring systemic treatment in our study group received it promptly, within about three weeks of diagnosis or surgery. The National Institute of Oncology serves as a reference center with an established Breast Unit. A multidisciplinary team, comprising a pathologist, radiologist, surgical oncologist, clinical oncologist, and radiotherapist, collaborates make therapeutic decisions for all patients with BC. Priority is given to the treatment of patients with high-risk factors, in accordance with European recommendations/guidelines (ESMO, St. Gallen 2013), which can be adopted by any oncology center (39, 54).

On the other hand, patients with Br-NENs should ideally be treated at Breast Units, which unfortunately are not available in every oncology center. Paradoxically, referring patients with a new diagnosis of Br-NEN from small hospitals to reference oncology centers or breast units, and delaying the start of therapy, may contribute to the poor prognosis in this group of patients.

### 4.1 Study limitations

The main limitation of the research is small patient cohort. This study is also limited by being conducted at a single center. However, the Maria Sklodowska-Curie National Research Institute of Oncology, Kraków Branch, has a large Breast Cancer Unit. An additional limitation is that the incidence of Br-NENs is suggested to be related to other factors, such as oral contraceptive use or early menarche (8). Unfortunately, this data was uncommonly available in our patients’ medical records. The third point to be mentioned is that our institution did not perform neuroenolase (NSE) staining, which could aid in diagnosis and has been utilized by other authors.

### 4.2 Primary strengths and attributes of the study

Br-NENs is a population often underrepresented in the literature due to its rarity. Only a few case series of patients with primary breast neuroendocrine malignancies have been published in the literature to date. Therefore, our cohort of patients remains one of the largest published so far, with data gathered about all types of treatment received (including systemic treatment, surgery, and radiotherapy). One notable strength of this research is the elucidation of the discrepancy in prognosis and risk of disease dissemination observed in various case series. This discrepancy underscores the importance of consistent definitions and inclusion criteria in research on Br-NENs. Furthermore, the implementation of standard therapeutic interventions, including surgery, radiotherapy, and systemic treatments tailored to individual patient profiles, demonstrates a pragmatic approach to managing this complex disease.

Importantly, the identification of platinum-based chemotherapy as a promising treatment option for poorly differentiated Br-NENs with high Ki67 levels adds to the armamentarium of therapeutic strategies available for clinicians. We underscore the need for prospective clinical trials to further explore the efficacy and safety of numerous coming options also in this patient population (43). Clinical trials are indispensable in the realm of different types of BC, as newer forms of treatment continue to emerge in the market (43). However, it is essential to note that clinical trials often exclude frail patients, and there is a pressing need for real-life data to complement these findings (55).

## 5 Conclusions

Br-NEN is an extremely heterogeneous and rare entity, with limited data available in the published literature and a few modifications in diagnostic classifications over the last few years. Although it is commonly suggested that NE differentiation worsens BC prognosis, this was not observed in our cohort. Likely, a significant discrepancy between published case series regarding prognosis and risk of disease dissemination, depends on the initially adopted definition of neuroendocrine breast neoplasms in the study’s inclusion criteria. This discrepancy is partially due to the rare occurrence of these cancers, frequent changes in classification, and the heterogeneity within this patient population. Patients with Br-NENs treated at our Cancer Centre received standard therapy, including surgery, radiotherapy, and systemic treatment. For those with poorly differentiated Br-NENs with high Ki67, platinum-based regimens were prescribed, leading to good responses. Prospective clinical trials should be planned for this population to obtain better knowledge about more effective treatment strategies.

## Data availability statement

The raw data supporting the conclusions of this article will be made available by the authors, without undue reservation.

## Ethics statement

The studies involving humans were approved by The Maria Sklodowska-Curie National Research Institute of Oncology Branch Krakow Ethical Committee approval was obtained (decision no. 3/2023). The studies were conducted in accordance with the local legislation and institutional requirements. The ethics committee/institutional review board waived the requirement of written informed consent for participation from the participants or the participants’ legal guardians/next of kin because Due to retrospective nature of the study written informed consents were not obtained from the patients as per Ethical Committee decision.

## Author contributions

The study’s conception and design were contributed by MP, MZ, JR, AA and JM. MP and JM searched the Cancer Center registry system. MP, AP, RP-M and AG-W organized the database and collected patients’ data, while MP, JM and AR carried out the statistical analysis. MP was responsible for Ethical Committee approval. The first draft of the manuscript was written by MP, and manuscript sections were written by AA, AM-M, RP-M, AG-W, AR, JM, figure was prepared by AA. All authors contributed to the article and approved the submitted version.
