# Supplementary material for: Non-metastatic primary neuroendocrine neoplasms of the breast: a reference cancer center’s experience of a heterogenous entity
Source: Front Endocrinol (Lausanne). 2024 May 10;15:1217495. doi: 10.3389/fendo.2024.1217495 (PMC11116701; doi:10.3389/fendo.2024.1217495)
Supplement: Supplementary file 1 [file Table_1.docx]

**Table S1.** Summary of presented in the literature case reports of neuroendocrine carcinomas of the breast with selected immunohistochemical features, applied treatment modalities and outcome (n=36 cases).

| Age/Sex | Histopathologic features | IHC | N+ | M+ | Surgical Treatment | Systemic treatment | Outcome | Ref |
| --- | --- | --- | --- | --- | --- | --- | --- | --- |
| 54/W | Neuroendocrine neoplasm with a signet ring feature | ER (-) HER2 (+) Syn +/- | Yes | No | Radical mastectomy with axillary lymph node dissection | Adjuvant chemotherapy 4 cycles ddAC $\to$ 12 cycles PXL + trastuzumab, pertuzumab 12 mo + AI 5 years | Follow-up 9 months | [1] |
| 71/W | Primary NEC of the breast | ER(+) PR (+)  HER2 (-) Syn (+) Chr (+) | No | No | Radical mastectomy with axillary lymph node dissection | Adjuvant endocrine therapy- AI 2 years | Follow-up 21 months | [2] |
| 48/W | Malignant invasive breast neoplasm suggesting NEC | Syn (+) Chr (+) | Yes | No | Lumpectomy and axillary lymph node dissection (at baseline) | Not applied | Lost to follow-up | [2] |
|  |  | ER(+) PR (+) HER2 (-) | Yes | Yes (after 1y) | Not applied | Palliative chemotherapy 1^st^ line: EC 8 cycles$\to$TAM 2^nd^ line: PXL+CBDDP 6 cycles $\to$ IA | Partial response |  |
| 65/W | Mixed invasive micropapillary and neuroendocrine mammary neoplasm | ER(+) PR (+) HER2 (-) Syn (+) Chr (+) | Yes | No | Radical mastectomy with axillary lymph node dissection | Adjuvant endocrine therapy AI 1 year and continues | Follow-up 12 mo | [3] |
| 64/M | Epithelial neoplasm with neuroendocrine differentiation | ER(+) PR (+) HER2 (-) Syn (+) Chr (+) CK (+) | Yes | No | Wide radical excision of the axillary tumor and axillary lymph node dissection | Adjuvant chemotherapy TC 4 cycles $\to$ TAM 1 year and continues | Follow-up 12 mo | [4] |
| 58/W | Well-differentiated NEC | ER(+) PR (+) HER2 (-) Syn (+) Chr (+) CK7 (+) CK20 (-) | No | No | Lumpectomy with SLNB | Not applied | NED | [5] |
| 62/W | DCIS and invasive mammary carcinoma with neuroendocrine and focal mucinous features | ER(+) PR (+) HER2 (-) Syn (+) | No | No data | Lumpectomy with SLNB | Not applied | NED | [5] |
| 34/W | Primary breast neuroendocrine tumor (solid NEC) | ER(+) PR (+) Chr (+) | No | No | Breast conserving surgery | Not applied | No data | [6] |
| 34/W | Primary NEC of the breast | ER(+) PR (+) CK7 (+) Syn (+) Chr (+) NSE(+) | No | No | Radical mastectomy with axillary lymph node dissection | Adjuvant chemotherapy FAC$\to$TAM | Follow-up 6 months | [7] |
| 51/W | Alveolar-type invasive primary neuroendocrine carcinoma of the breast  with invasive ductal carcinoma | ER(+) PR (-) HER2 (-) CK7 (+) Syn (+) Chr (+) | No | No | Radical mastectomy | NCT 4 cycles AC $\to$ surgery → Adjuvant chemotherapy$3 cycles$DDP+V16 $\to3 cycles$PXL+CBDDP $\to$ TAM $\to$ AI | M+ after 13 mo | [8] |
| 56/W | Small-cell undifferentiated carcinoma of the breast (breast cancer with endocrine differentiation) | Syn (+) Chr (-) | No | No | Breast-conserving surgery with axillary lymph node dissection | Adjuvant chemotherapy 5 cycles CMF → toremifene and continues | Follow-up 26 mo | [9] |
| 71/W | Carcinoid tumor | ER(+) PR (+) Syn (+) Chr (+) NSE(+) | No | No | Radical mastectomy with axillary lymph node dissection | Adjuvant endocrine therapy- toremifene and continues | Follow-up 12 mo | [9] |
| 64/W | Small cell neuroendocrine carcinoma of the breast | NSE(+) | No | Yes | No data | No data | No data | [10] |
| 29/W | Bilateral primary breast NEC of the carcinoid tumor type | ER(+) PR (+) HER2 (-) Syn (+) Chr (+) NSE(+) CK7 (+) | No | No | Lumpectomy | Adjuvant chemotherapy 3 cycles CEF+3 cycles TXL→ toremifene and continues | Follow-up 20 mo | [11] |
| 75/W | NEC with a carcinoid-like pattern | Syn (+) Chr (+) NSE(+) | No | No | Breast conserving surgery | No data | No data | [12] |
| 64/W | Primary solid NEC of the breast | ER(+) PR (+) Syn (+) Chr (+) | No | No | Radical mastectomy with axillary lymph node dissection | Adjuvant chemotherapy | Follow-up 8 mo | [13] |
| 27/W | Primary large-cell neuroendocrine carcinoma of the breast | Syn (+) Chr (+) NSE(+) | No | No | Breast-conserving surgery and axillary lymph node dissection | Adjuvant chemotherapy | Follow-up 18 mo | [14] |
| 40/W | Invasive NEC with a relatively prominent intraductal component | ER(+) PR (+) HER2(-) Syn(+) Chr(+) CD56(+) | No | No | Radical mastectomy with SLNB | Adjuvant endocrine therapy- AI | Follow-up 36 mo | [15] |
| 40/W | Primary NEC of the breast | Syn (+) | Yes | No | Radical mastectomy with axillary lymph node dissection | No data | No data | [16] |
| 53/W | Infiltrating ductal carcinoma. 3 years later primary NEC of the breast. | ER (-) PR (-) HER2 (-) TTF1(-) Syn (+) Chr (+) | Yes | Yes | No data | No data | No data | [17] |
| 83/M | Argyrophilic breast carcinoma with neuroendocrine differentiation | ER (-) Syn (+) Chr (+) | No | No | Simple mastectomy | No data | OS 7y dead of other disease | [18] |
| 68/W | Neuroendocrine primary small cell carcinoma of the breast | ER (-) PR (-) Chr (+) | No | No | Radical mastectomy with axillary lymph node dissection | No applied | OS 21mo | [19] |
|  |  |  | Yes | Yes after 1y | No applied | Adjuvant chemotherapy AC+VP16 |  |  |
| 56/W | Small cell (oat cell) carcinoma of the breast | ER (-) PR (-) Syn (+) Chr (+) | ? | No | Radical mastectomy with axillary lymph node dissection | No applied | Follow-up 4y | [20] |
| 60/W | Primary neuroendocrine small cell carcinoma of the breast | Syn (+) Chr (+) NSE (+) | Yes | No | Radical mastectomy with axillary lymph node dissection | NCT 3 cycles FEC → surgery → adjuvant chemotherapy 4 cycles FEC/CBDDP+VP16 | Follow-up 6 mo | [21] |
| 41/W | Primary small cell (oat cell) carcinoma of the breast | ER (-) Chr (+) NSE (+) CK (+) | No | No | Radical mastectomy with axillary lymph node dissection | Adjuvant chemotherapy 6 cycles CMF | Follow-up 16 mo | [22] |
| 46/W | Primary small cell carcinoma of the breast | ER (-) PR (+)\| Chr (-) NSE(-) | No | No | Lumpectomy | Adjuvant chemotherapy 6 cycles DDP +VP16 | Follow-up 9 mo | [23] |
| 62/W | Primary small cell carcinoma of the breast | ER (-) PR (-) HER2 (-) NSE(+) E-cadherin(-) | ? | No | Radical mastectomy with axillary lymph node dissection | No data | No data | [24] |
| 71/W | Primary NEC of the breast | ER (+) PR (+) HER2 (-) Syn (+) Chr (+) NSE(+) CK(+) | No | No | Radical mastectomy with axillary lymph node dissection | Adjuvant endocrine therapy TAM and continues | Follow-up 12 mo | [25] |
| 58/W | Primary carcinoma of breast with neuro-endocrinesmall-cell features | ER (-) PR (-) HER2 (-) Chr (-) NSE(+) | Yes | No | Wide local excision with axillary lymph node dissection | Adjuvant chemotherapy Adriamicin + DDP | Follow-up 18 mo | [26] |
| 59/W | Infiltrative breast carcinoma with morphological neuroendocrine features | ER (+) PR (+) Chr (+) | No | No | Radical mastectomy | Adjuvant endocrine therapy TAM 3y | Distant RFS 11y | [27] |
|  | Primary NEC of the breast | ER (+) PR (-) Syn (+) Chr (+) NSE(+) | No | Yes | Metastasectomy | Palliative chemotherapy 1^st^ line Epirubicin → AI | Follow-up 3y |  |
| 31/W | Primary small-cell neuroendocrine carcinoma | ER (-) PR (-) HER2 (-) Syn (+) Chr (+) NSE(+) CD56(+) | Yes | Yes 5 weeks afer surgery | Radical mastectomy with axillary lymph node dissection | NCT 3 cycles TXL + Adriamycin →surgery → palliative chemotherapy 1^st^ line DDP + irinotecan | OS 9 mo | [28] |
| 64/W | Primary solid NEC | ER (+) PR (+) Syn (+) Chr (+) | No | No | Radical mastectomy with axillary lymph node dissection | Adjuvant chemotherapy | Follow-up 8mo | [29] |
| 51/W | Neuroendocrine small cell carcinoma of the breast | ER (-) PR (-) HER2 (-) Syn (+) Chr (+) CK8(+) | No | No | Radical mastectomy with axillary lymph node dissection | Adjuvant chemotherapy 6 cycles PXL | Follow-up 1y | [30] |
| 61/W | Primary small cell carcinoma of the breast | ER (-) PR (-) HER2 (-) Syn (+) Chr (+) CD56(+) TTF-1(+) | Yes | Yes 4 weeks after surgery | Radical mastectomy with axillary lymph node dissection | Palliative chemotherapy 1^st^ line CBDDP+VP16 | OS 3mo | [31] |
| 72/M | Neuroendocrine DCIS coexistent with a background of neuroendocrine cell hyperplasia | ER (+) PR (+) HER2 (-) Syn (+) CD56(+) | No | No | Partial mastectomy with SLNB | No data | No data | [32] |
| 52/M | Small cell NEC (oat cell) of the breast | NSE(+) CK(-) | Yes | Yes | Not applied | Palliative chemotherapy | OS 14 mo | [33] |

Abbreviations: AI- aromatase inhibitor, CBDDP- carboplatin, CEF- cyclophosphamide + epirubicin + fluorouracil, Chr- chromogranin, CK- cytokeratin, CMF-cyclophosphamide, methotrexate and fluorouracyl, DCIS- ductal carcinoma in situ, ddAC- dose-dense doxorubicin + cyclophosphamide, DDP-cisplatin, EC- epirubicin + cyclophosphamide, ER- estrogen receptor, HER2- human epidermal growth factor receptor 2, IHC- immunohistochemistry, IQR -interquartile range, M+ presence of distance metastases, M-men, mOS/OS- median/overall survival, mo-months, mRFS- median relapse-free survival, n- number of cases, N+ presence of metastases in regional lymph nodes, NCT- neoadjuvant chemotherapy, NEC- neuroendocrine carcinoma, NED - no evidence of disease, NSE- neuron specific enolase, Syn- synaptophysin, PR- progesterone receptor, PXL - paclitaxel, Ref- reference, SLNB- sentinel lymph node biopsy, TAM- tamoxifen, TC- docetaxel + cyclophosphamide, TTF1- thyroid transcriptional factor-1, TXL- docetaxel, V16- etoposide, W-women, y-years

References for Table s1:

1. Li Y, Cao Y, Wu X, Liu R, Wang K. HER-2-positive primary neuroendocrine neoplasms of the breast with signet ring feature: A case report and review of literature. Front Oncol [Internet]. 2022 Dec 12 [cited 2023 Jan 5];12:1029007. Available from: /pmc/articles/PMC9791177/

2. Hejjane L, Oualla K, Bouchbika Z, Bourhafour M, Lhlou Mimi A, Boubacar E, et al. Primary neuroendocrine tumors of the breast: two case reports and review of the literature. J Med Case Rep [Internet]. 2020 Mar 10 [cited 2023 Jan 5];14(1). Available from: /pmc/articles/PMC7065345/

3. Kawasaki T, Tashima T, Muramatsu C, Fujimoto A, Usami Y, Kodama H, et al. Neuroendocrine tumor of the breast showing invasive micropapillary features and multiple lymph node metastases. Cancer Rep [Internet]. 2022 [cited 2023 Jan 5];e1775. Available from: https://onlinelibrary.wiley.com/doi/full/10.1002/cnr2.1775

4. Kimura K, Kawabata S, Oku H, Ikari A, Tominaga T, Takai S, et al. Breast neuroendocrine tumor arising in the axilla of a man: a case report. J Med Case Rep [Internet]. 2022 [cited 2023 Jan 5];16:1–5. Available from: /pmc/articles/PMC9759905/

5. Collado-Mesa F, Net JM, Klevos GA, Yepes MM. Primary neuroendocrine carcinoma of the breast: report of 2 cases and literature review. Radiol Case Rep [Internet]. 2017 Mar 1 [cited 2023 Jan 5];12(1):1–12. Available from: https://pubmed.ncbi.nlm.nih.gov/28228868/

6. Ogawa H, Nishio A, Satake H, Naganawa S, Imai T, Sawaki M, et al. Neuroendocrine tumor in the breast. Radiation Medicine - Medical Imaging and Radiation Oncology [Internet]. 2008 Jan 31 [cited 2023 Jan 5];26(1):28–32. Available from: https://link.springer.com/article/10.1007/s11604-007-0182-y

7. Murthy V, Geethamala K, Kumar B, Sudharao M. Primary Neuroendocrine Carcinoma of Breast: A Rare Case Report. Ann Med Health Sci Res [Internet]. 2013 [cited 2023 Jan 5];3(Suppl1):S35. Available from: /pmc/articles/PMC3853605/

8. Angarita FA, Rodríguez JL, Meek E, Sánchez JO, Tawil M, Torregrosa L. Locally-advanced primary neuroendocrine carcinoma of the breast: case report and review of the literature. World J Surg Oncol [Internet]. 2013 Jun 5 [cited 2023 Jan 5];11:128. Available from: /pmc/articles/PMC3682896/

9. Ajisaka H, Maeda K, Miwa A, Yamamoto K. Breast Cancer with Endocrine Differentiation: Report of Two Cases Showing Different Histologic Patterns. Surg Today [Internet]. 2003 [cited 2023 Jan 5];33(12):909–12. Available from: https://link.springer.com/article/10.1007/s00595-003-2612-5

10. An JK, Woo JJ, Kang JH, Kim EK. Small-cell neuroendocrine carcinoma of the breast. J Korean Surg Soc [Internet]. 2012 Feb [cited 2023 Jan 5];82(2):116–9. Available from: https://pubmed.ncbi.nlm.nih.gov/22347714/

11. Zhang JY, Chen WJ. Bilateral primary breast neuroendocrine carcinoma in a young woman: report of a case. Surg Today [Internet]. 2011 Nov [cited 2023 Jan 5];41(11):1575–8. Available from: https://pubmed.ncbi.nlm.nih.gov/21969166/

12. Valentim MH, Monteiro V, Marques JC. Primary neuroendocrine breast carcinoma: a case report and literature review. Radiol Bras [Internet]. 2014 [cited 2023 Jan 5];47(2):125–7. Available from: https://pubmed.ncbi.nlm.nih.gov/25741062/

13. Stita W, Trabelsi A, Gharbi O, Mokni M, Korbi S. Primary solid neuroendocrine carcinoma of the breast. Canadian Journal of Surgery [Internet]. 2009 [cited 2023 Jan 5];52(6):E289. Available from: /pmc/articles/PMC2792409/

14. Kim JW, Woo OH, Cho KR, Seo BK, Yong HS, Kim A, et al. Primary large cell neuroendocrine carcinoma of the breast: radiologic and pathologic findings. J Korean Med Sci [Internet]. 2008 Dec [cited 2023 Jan 5];23(6):1118–20. Available from: https://pubmed.ncbi.nlm.nih.gov/19119462/

15. Fujimoto Y, Yagyu R, Murase K, Kawajiri H, Ohtani H, Arimoto Y, et al. A case of solid neuroendocrine carcinoma of the breast in a 40-year-old woman. Breast Cancer [Internet]. 2007 Apr 1 [cited 2023 Jan 6];14(2):250–3. Available from: https://link.springer.com/article/10.2325/jbcs.889

16. Akhtar K, Zaheer S, Ahmad S, Hassan M. Primary neuroendocrine carcinoma of the breast. Indian J Pathol Microbiol [Internet]. 2009 Jan 1 [cited 2023 Jan 6];52(1):71. Available from: https://www.ijpmonline.org/article.asp?issn=0377-4929;year=2009;volume=52;issue=1;spage=71;epage=73;aulast=Akhtar

17. McIntire M, Siziopikou K, Patil J, Gattuso P. Synchronous metastases to the liver and pancreas from a primary neuroendocrine carcinoma of the breast diagnosed by fine-needle aspiration. Diagn Cytopathol [Internet]. 2008 Jan [cited 2023 Jan 6];36(1):54–7. Available from: https://pubmed.ncbi.nlm.nih.gov/18064696/

18. Papotti M, Tanda F, Bussolati G, Pugno F, Bosincu L, Massareli G. Intriguing Case: Argyrophilic Neuroendocrine Carcinoma of the Male Breast. http://dx.doi.org/103109/01913129309015404 [Internet]. 2009 [cited 2023 Jan 6];17(1):115–20. Available from: https://www.tandfonline.com/doi/abs/10.3109/01913129309015404

19. Francois A, Chatikhine VA, Chevallier B, Guo Sheng Ren, Berry M, Chevrier A, et al. Neuroendocrine primary small cell carcinoma of the breast. Report of a case and review of the literature. Am J Clin Oncol [Internet]. 1995 [cited 2023 Jan 6];18(2):133–8. Available from: https://pubmed.ncbi.nlm.nih.gov/7534975/

20. Fukunaga M, Ushigome S. Small cell (oat cell) carcinoma of the breast. Pathol Int [Internet]. 1998 Sep 1 [cited 2023 Jan 6];48(9):744–8. Available from: https://onlinelibrary.wiley.com/doi/full/10.1111/j.1440-1827.1998.tb03976.x

21. Samli B, Celik S, Evrensel T, Orhan B, Tasdelen I. Primary Neuroendocrine Small Cell Carcinomaof the Breast. Arch Pathol Lab Med [Internet]. 2000 Feb 1 [cited 2023 Jan 6];124(2):296–8. Available from: https://meridian.allenpress.com/aplm/article/124/2/296/452540/Primary-Neuroendocrine-Small-Cell-Carcinomaof-the

22. Yamasaki T, Shimazaki H, Aida S, Tamai S, Tamaki K, Hiraide H, et al. Primary small cell (oat cell) carcinoma of the breast: Report of a case and review of the literature. Pathol Int [Internet]. 2000 Nov 1 [cited 2023 Jan 6];50(11):914–8. Available from: https://onlinelibrary.wiley.com/doi/full/10.1046/j.1440-1827.2000.01126.x

23. Salmo EN, Connolly CE. Primary small cell carcinoma of the breast: report of a case and review of the literature. Histopathology [Internet]. 2001 Mar 1 [cited 2023 Jan 6];38(3):277–8. Available from: https://onlinelibrary.wiley.com/doi/full/10.1046/j.1365-2559.2001.01068.x

24. Bergman S, Hoda SA, Geisinger KR, Creager AJ, Trupiano JK. E-cadherin-negative primary small cell carcinoma of the breast. Report of a case and review of the literature. Am J Clin Pathol [Internet]. 2004 Jan 1 [cited 2023 Jan 6];121(1):117–21. Available from: https://pubmed.ncbi.nlm.nih.gov/14750249/

25. Jochems L, Tjalma WAA. Primary small cell neuroendocrine tumour of the breast. European Journal of Obstetrics and Gynecology and Reproductive Biology [Internet]. 2004 Aug 10 [cited 2023 Jan 6];115(2):231–3. Available from: http://www.ejog.org/article/S0301211503006444/fulltext

26. Sridhar P, Matey P, Aluwihare N. Primary carcinoma of breast with small-cell differentiation. Breast [Internet]. 2004 Apr 1 [cited 2023 Jan 6];13(2):149–51. Available from: http://www.thebreastonline.com/article/S096097760300167X/fulltext

27. Berruti A, Saini A, Leonardo E, Cappia S, Borasio P, Dogliotti L. Management of neuroendocrine differentiated breast carcinoma. Breast [Internet]. 2004 [cited 2023 Jan 6];13(6):527–9. Available from: https://pubmed.ncbi.nlm.nih.gov/15563864/

28. Kinoshita S, Hirano A, Komine K, Kobayashi S, Kyoda S, Takeyama H, et al. Primary small-cell neuroendocrine carcinoma of the breast: report of a case. Surg Today [Internet]. 2008 Aug [cited 2023 Jan 6];38(8):734–8. Available from: https://pubmed.ncbi.nlm.nih.gov/18668318/

29. Stita W, Trabelsi A, Gharbi O, Mokni M, Korbi S. Primary solid neuroendocrine carcinoma of the breast. Canadian Journal of Surgery [Internet]. 2009 [cited 2023 Jan 6];52(6):E289. Available from: /pmc/articles/PMC2792409/

30. Yamaguchi R, Tanaka M, Otsuka H, Yamaguchi M, Kaneko Y, Fukushima T, et al. Neuroendocrine small cell carcinoma of the breast: report of a case. Med Mol Morphol [Internet]. 2009 Mar [cited 2023 Jan 6];42(1):58–61. Available from: https://pubmed.ncbi.nlm.nih.gov/19294494/

31. Christie M, Chin-Lenn L, Watts MM, Tsui AE, Buchanan MR. Primary small cell carcinoma of the breast with TTF-1 and neuroendocrine marker expressing carcinoma in situ. Int J Clin Exp Pathol [Internet]. 2010 [cited 2023 Jan 6];3(6):629. Available from: /pmc/articles/PMC2907125/

32. Miura K, Nasu H, Ogura H. Double neuroendocrine ductal carcinomas in situ coexisting with a background of diffuse idiopathic neuroendocrine cell hyperplasia of breast: a case report and hypothesis of neuroendocrine tumor development. Pathol Int [Internet]. 2012 May [cited 2023 Jan 8];62(5):331–4. Available from: https://pubmed.ncbi.nlm.nih.gov/22524661/

33. Jundt G, Schulz A, Heitz PU, Osborn M. Small cell neuroendocrine (oat cell) carcinoma of the male breast. Immunocytochemical and ultrastructural investigations. Virchows Arch A Pathol Anat Histopathol [Internet]. 1984 Jun [cited 2023 Jan 8];404(2):213–21. Available from: https://pubmed.ncbi.nlm.nih.gov/6091325/
